# Modeling Natural Photic Entrainment in a Subterranean Rodent (*Ctenomys* aff. *knighti*), the Tuco-Tuco

**DOI:** 10.1371/journal.pone.0068243

**Published:** 2013-07-10

**Authors:** Danilo E. F. L. Flôres, Barbara M. Tomotani, Patricia Tachinardi, Gisele A. Oda, Veronica S. Valentinuzzi

**Affiliations:** 1 Departamento de Fisiologia, Instituto de Biociências, Universidade de São Paulo, São Paulo, São Paulo, Brazil; 2 Centro Regional de Investigaciones Científicas y Transferencia Tecnológica-CRILAR, Entre Rios y Mendoza s/n, (5301) Anillaco, La Rioja, Argentina; Morehouse School of Medicine, United States of America

## Abstract

Subterranean rodents spend most of the day inside underground tunnels, where there is little daily change in environmental variables. Our observations of tuco-tucos (*Ctenomys* aff. *knighti*) in a field enclosure indicated that these animals perceive the aboveground light-dark cycle by several bouts of light-exposure at irregular times during the light hours of the day. To assess whether such light-dark pattern acts as an entraining agent of the circadian clock, we first constructed in laboratory the Phase Response Curve for 1 h light-pulses (1000lux). Its shape is qualitatively similar to other curves reported in the literature and to our knowledge it is the first Phase Response Curve of a subterranean rodent. Computer simulations were performed with a non-linear limit-cycle oscillator subjected to a simple model of the light regimen experienced by tuco-tucos. Results showed that synchronization is achieved even by a simple regimen of a single daily light pulse scattered uniformly along the light hours of the day. Natural entrainment studies benefit from integrated laboratory, field and computational approaches.

## Introduction

Subterranean species are interesting for circadian entrainment studies because they live in underground burrows where 24 h environmental light/dark (LD) cycles are presumed to be attenuated. Laboratory studies with solitary tuco-tucos from La Rioja, Argentina (*Ctenomys* aff. *knighti*) have shown that this species is precisely entrainable by LD 12∶12 (12 hours of light and 12 hours of dark cycles), being dark-active under this condition [1]. In field enclosures, individuals of this species emerge to the surface and expose themselves to light during brief episodes of aboveground foraging and soil removal activities. Moreover, rhythms of all animals transferred from the field to constant darkness in the laboratory display 24 h period-“aftereffects” [2], indicating that tuco-tucos had been previously entrained in the field [3].

**Figure 1:**
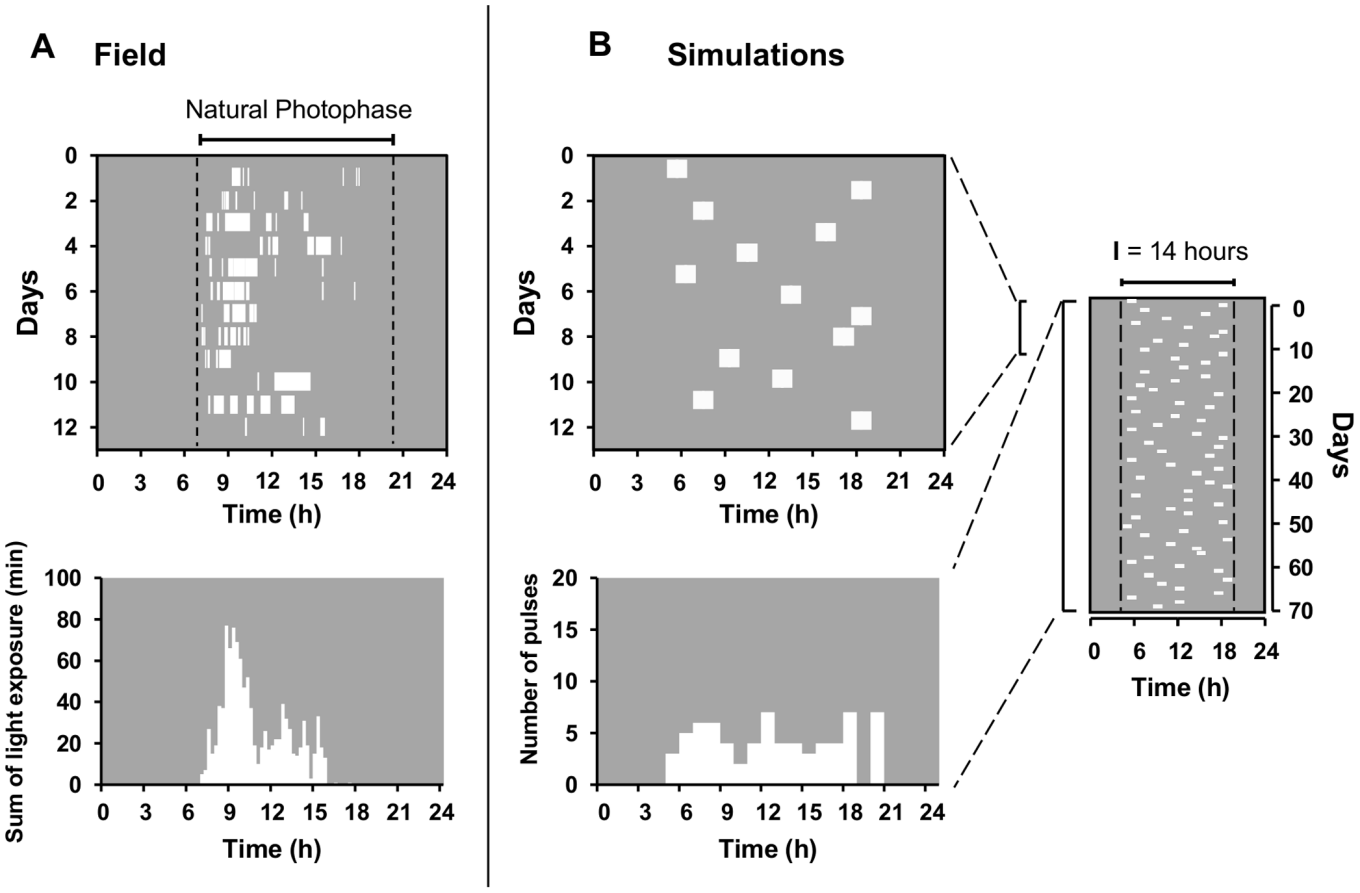
Light-dark regimen experienced by a tuco-tuco in the field enclosure and a simplified model reproduced in computer simulations. A: Field data obtained by continuous observations of an animal in a semi-natural enclosure for 12 days, during the light-hours of the day (modified from Tomotani et al., 2012) [3]. In the upper graphic (actogram) white marks indicate the light-exposure episodes (aboveground activity) superimposed on a darkness background (animal underground and/or at night). The time of light exposure in the field varied from day to day. It was however more probable at the beginning of the day, as revealed by the sum of exposure from all days (lower graphic). B: Our simplified model of light-exposure, consisting of a single 1 h-light-pulse per day, whose time changed every day (upper graphic) in a uniformly random fashion (lower graphic), but always restricted to a time-window I (graphic on the right).

Direct observation of individual tuco-tucos in field enclosures revealed that these subterranean animals do not expose themselves to the full time-length of the daylight [3]. Emergence episodes last from seconds to around one hour and occur at variable times of the day, with a phase of higher probability (Fig. 1A). To approach field entrainment of tuco-tucos under this particular light regimen, we performed laboratory experiments and mathematical modeling.

In the laboratory, we first verified whether cycles consisting of a darkness background with one light pulse every 24 hours, known as T24 single-pulse T-cycles [4], [5] could entrain the circadian oscillator of tuco-tucos. Then we constructed the light Phase Response Curve (PRC) [6]–[8] of tuco-tucos. This is one way of assessing the daily variation of the circadian oscillator sensitivity to light stimuli, through the ensued differential phase shifts; to our knowledge, this was not done before in subterranean rodents. A different approach is to measure varying *fos* expression in the circadian clock in response to light stimuli throughout the day. Such variation was shown in subterranean mole rats [9].

Computer simulations were performed with a limit-cycle oscillator displaying a PRC that has qualitative features of the experimentally obtained curve. We submitted this oscillator to a light regimen that mimics the tuco-tucós daily exposure pattern in the field, to investigate the minimal elements of a random light-exposure regimen that can sustain entrainment of the circadian oscillator. Natural entrainment studies [10] benefit from integrated field, laboratory and computational approaches.

## Materials and Methods

### Ethics Statement

The capture and laboratory experimentation protocols were approved and authorized by the Legal and Technical board (*Oficina de Técnica Legal*) of the Environmental Department of La Rioja (*Secretaria de Ambiente, Ministerio de Producción y Desarrollo Local*), permit number 062-08. Every procedure in this study followed the guidelines of the American Society of Mammalogists for animal care and handling [11].

### Animals and Housing

The study species first identified as *Ctenomys knighti* is now undergoing a taxonomical analysis. Biological samples were sent to the *Colección Mastozoológica del IADIZA, Mendoza* (specimens CMI 07458 and 07459). For further details see Tomotani et al. [3].

Animals were captured using PVC tube traps, in the region of Anillaco, Castro Barros Department, province of La Rioja, Argentina (28°48′S; 66°56′W; 1,350 m). A total of 13 females and 11 males was used in the experiments. Average weight upon capture was 140.4±33.4 g. They were housed individually in acrylic running-wheel cages (53×29×27 cm) connected to a continuous recording system (Simonetta System, Universidad Nacional de Quilmes, Buenos Aires, Argentina), for the assessment of their activity-rest rhythms through all the experiments.

Cages were cleaned weekly and animals were fed daily at random times, with carrots, sweet potatoes, lettuce, sunflower seeds and rabbit pellets. Subterranean rodents do not drink free water [12]. Room temperature was kept at 23±2°C. A background dim light (<5 lux) remained turned on along the course of all experiments to allow cleaning and feeding, even during constant darkness (DD) conditions. It was provided by two incandescent red lamps (Philips 40/25 W) connected to a dimmer (200 W, Teclastar Milano, San Martín, Buenos Aires, Argentina). “Light” and “dark” conditions consisted of turning on/off fluorescent bulbs (around 1000 lux, 36W, 1.2 m-long Cool Day Light, OSRAM L) over the background dim light. The representation **LD X:Y** is used to indicate light-dark cycles of X hours of light and Y hours of darkness.

### Experiments – Entrainment to a T-24 Cycle and Phase Response Curve

To assess the daily varying sensitivity of the light-entrainable oscillator of the tuco-tucos to light-stimuli we measured their PRCs.

For this purpose, 23 individuals were first submitted to 24 h period single pulse T-cycles consisting of a 1-hour light pulse every 24 h (LD 1∶23, L_ = _1000lux) during a 30-day interval. Synchronization to the T-24 cycle was verified in actograms build in the software El Temps (A. Díez-Noguera, Universitat de Barcelona, 1999) and assessed with a χ^2^periodogram analysis [13] in the software ClockLab (Actimetrics, Wilmette, IL). When synchronization was confirmed, we calculated the phase-relationship between activity onset and offset and the start of the 1-hour light-pulse in the last 5 days under synchronization. Calculations were performed in the software R [14].

Thereafter, animals were released into DD for PRC construction. After 30 days all individuals received a one-hour lights-on stimulus (1000 lux) simultaneously at a programmed time. This was followed by another 30 days in constant darkness. This entire schedule (LD 1∶23, DD, light pulse, DD) was repeated 3 times. Due to individual variations in phase and period, light pulses were administered in a different *subjective phase* for each animal, within each of the 3 repeated schedules.

The subjective phase of the pulse is measured in a relative daytime scale (circadian time, CT), based on the phase and period of the free-running activity-rest rhythm. By convention, activity onset is considered circadian time 12 (CT12) [8]. The duration of one circadian hour corresponds to the free-running period τ in hours (estimated for each individual by eye-fitting) divided by 24. The subjective circadian phase (CT) of the light pulse administration for each individual was thus calculated by measuring the interval, in circadian hours, from the time of the pulse to the reference phase CT12.

Activity-onsets were used as a phase-reference of the activity-rest rhythm. Hence, the phase of the rhythm before and after the light-pulse was recognized through eye-fitting of a straight line along successive activity onsets. For phase measurements after the light-pulse, we only considered the days in which the rhythm had already achieved a new steady state, i.e., after the “transient” cycles [15]. Phase shifts were then calculated from the difference in hours between the two fitted lines (pre-pulse and post-pulse) on the day of the light pulse. By convention, when the pulse shifts the phase of the rhythm to an earlier time, the value of this phase-advance is plotted as positive in the ordinate of the PRC. Conversely, phase-delays are plotted as negative values in the ordinate [8]. Data for phase-shifts is presented as mean ± standard deviation.

All calculations were performed in the software El Temps (A. Díez-Noguera, *Universitat de Barcelona*, 1999). Data of activity-rest rhythms of all the animals were plotted in actograms to allow visual analysis and eye-fitting of phase-reference lines for the measurement of phase-shifts. The free-running period (τ) values were calculated by the inclination of the pre-pulse eye-fitted lines used as phase-references.

Statistical analysis of the resulting PRC was made by grouping together the phase-shifts in 4 groups corresponding to 6 circadian hours each. Based on the Shapiro-Wilk test for normality of the residues, the Kruskal-Wallis test was chosen to verify significant difference among the 4 groups. Although some individuals were repeated across groups, Friedman test for repeated measures could not be used, because not all individuals were present in all compared groups. Finally, Wilcoxon test was used for group comparisons in pairs, using the Bonferoni correction for multiple comparisons. Statistical tests were performed in the software R [14] and the significance level was 0.05.

### Computer Simulations of a Field Entrainment Model

Computer simulations were performed to test the entrainment effectiveness of the irregular, natural light-exposure patterns (Fig. 1A) experienced by tuco-tucos in the field. A simple model of light regimen, which assumes randomness in light-exposure, was impinged on a model limit-cycle oscillator. The aim of the simulations was to evaluate the minimal regularity of a daily light-exposure pattern that can sustain entrainment of a circadian oscillator. The following simplified light regimen (Fig. 1B) was used as a starting point:

1. A single light pulse of fixed 1 hour duration per day;
2. Fixed light intensity, independent of the time of the light pulse;
3. Uniform randomness of light pulse occurrence along a restricted daily time interval.

The restricted time-interval (**I**) of pulse occurrence represents the natural light-phase (Fig. 1B right). By changing the duration of this light-phase interval **I**, we created regimens with different dispersion of the pulses along the 24 h day. For instance, **I**_ = _0 represents one pulse occurring every day at the same time, as a regular LD 1∶23 cycle. Accordingly, **I**_ = _2 hours represents one daily light-pulse occurring at a uniformly random time, restricted to a fixed 2-hour interval of the day. To generate uniformly distributed light pulse phases, we used Excel (2007) function RandBetween, which returns a random number within the fixed interval **I**, with all numbers with equal probability. This process was repeated 70 times to generate the simulated 70-day light regimens. Each regimen was built by increasing **I** to 4, 6, 8, 10, 12, 14, 16, 18 and 24 hours. Due to the extreme simplifications of this light regimen, loss of entrainment was expected to occur as soon as **I** were minimally increased.

The model limit-cycle oscillator used in the computer simulations displays a PRC with advances and delays [16], qualitatively similar to the experimental curve of tuco-tucos (Fig. S1). We used the limit-cycle oscillator developed by Pavlidis and Pittendrigh [17], which is defined by the following equations:
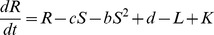


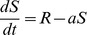



In these equations, *R* and *S* are the state variables and letters *a* to *d* are the four parameters, whose manipulation generates different oscillator configurations. *L* represents the light level; a single light pulse is mimicked by square-wave changes of *L* from baseline 0 to amplitude 1 (arbitrary unit) for the duration of the pulse. *K* is a small nonlinear term (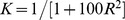) formulated by W.T. Kyner that helps preventing the *R* variable to approximate zero. We chose to use a standard configuration (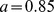, 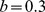, 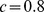, 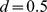), already employed in other works [16], [17]. Simulated locomotor activity occurred every time the *S* variable rose above a threshold value, which we set to two-thirds of the maximum amplitude of this variable, as explicitly shown in [16].

Simulations were performed using the software *Neurodynamix* [18]. For every simulated light-regimen, output rhythms of the model oscillator were plotted in actograms in the software El Temps and entrainment was evaluated with χ^2^periodogram analysis [13] in the software ClockLab. This procedure allowed both visual and statistical evaluations of the model oscillator entrainment to a 24-h period cycle. In addition, we calculated the day-to-day variability of activity onset phase of the oscillator, for all the simulated light regimens with the software R [14].

## Results

### Entrainment to a T-24 cycle

**Figure 2:**
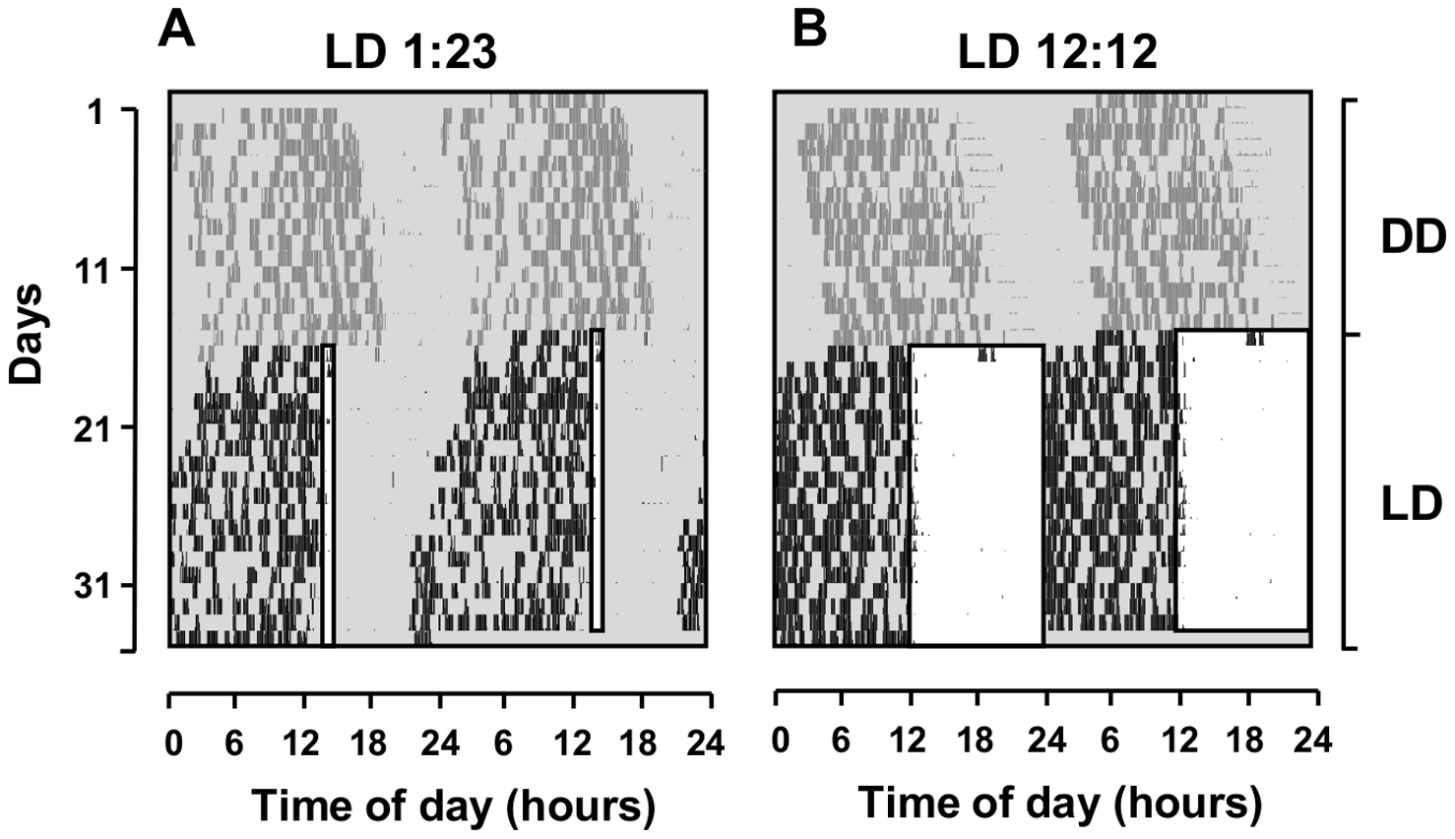
Synchronization of the wheel-running activity rhythm of a tuco-tuco by light dark cycles LD 1∶23 (A) and LD 12∶12 (B). The data is depicted in actograms, with times of running-wheel activity (black and dark-gray marks) represented along the days. Dark-gray marks represent free-running rhythms in the first 15 days in DD. White rectangles drawn in the graphics during the LD cycles represent the hours of lights-on. Both light-dark cycles LD 1∶23 and LD 12∶12 synchronize the rhythms of tuco-tucos to a 24 h-period. Figure B was modified from Valentinuzzi et al. (2009) [1].

A representative actogram (Fig. 2A) displays an animal’s free-running rhythm that is synchronized by the periodic 1 h-light pulses, with activity offset locked to the beginning of the pulse. Seventeen out of the 23 animals displayed this same pattern of entrainment, with activity offset within less than 1 hour of the beginning of the pulse: on average, activity offset preceded the pulse in 50 minutes _±_1 h and 32 minutes. The other animals did entrain (except for one individual in one LD trial) but with larger phase relationship to the light pulse. Four animals had offsets at 2 h9 min _±_47 min before the light pulse and one animal at 6 h20 min _±_2 h48 min for 4 trials. For comparison, the running-wheel activity rhythm of the same individual under LD12∶12 (L around 200 lux) is shown in Fig. 2B (modified from [1]). Under this exposure to a full photoperiod, daily activity is concentrated in the dark phase.

### Phase Response Curve

**Figure 3:**
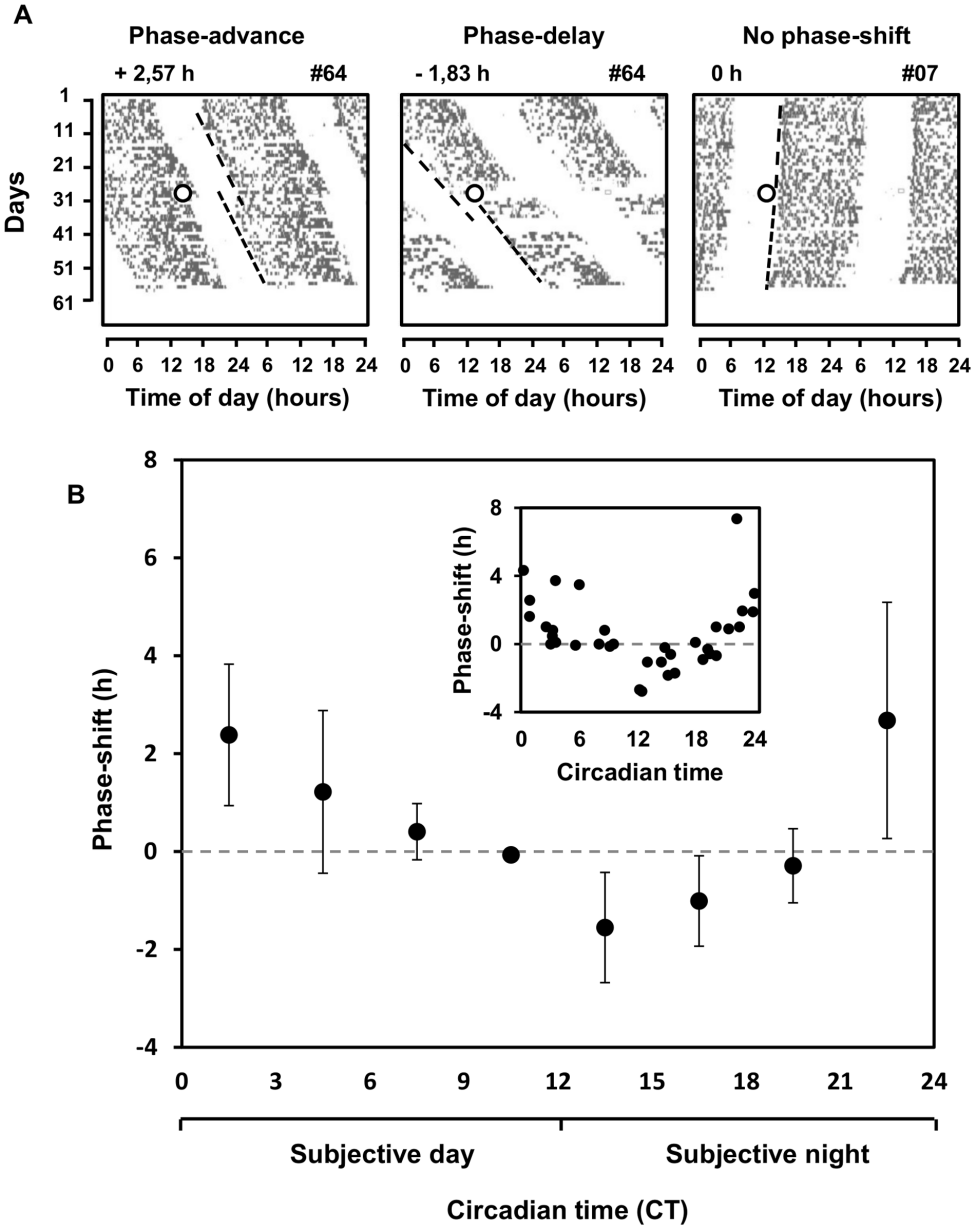
Photic Phase Response Curve (PRC) of the tuco-tuco. A: Representative phase-shifts in response to light-pulses. The actograms show three examples of free-running rhythms of tuco-tucos under DD conditions and different phase-shift responses of the rhythms elicited by a single 1 h-light-pulse (1000 lux) on day 28 (open circle). In each graph, the phase of activity onsets after the pulse (lower dashed line) is compared to the phase of activity onsets previous to the pulse (upper dashed line). In response the light stimuli, activity phase was either advanced (left graph), delayed (middle graph) or unshifted (right graph). Values on the upper left of each graph are quantifications, in hours, of the phase-shift responses. The upper right numbers are identification codes for individual animals. B: The magnitudes of phase-shift responses are plotted against the time of the applied light-pulse. The time axis is represented in a standardized time-scale (circadian time). Inset: single values for each of the phase-shift responses. Outer graph: mean phase-shifts ±1 standard deviation for every 3 CT’s. Both graphs illustrate the dependence of the magnitude and sign of phase-shift on the time of the pulse.

In response to the 1 h-light-stimuli, tuco-tucos presented phase dependent advances, delays or no phase-shift at all in the phase of their activity-rest rhythms, as can be seen in the representative actograms of Fig. 3A. The other actograms are available in Fig. S2. The resulting PRC is displayed in Fig. 3B.

Visual inspection of the data points and the means for every 3 CT (Fig. 3B) reveals that phase-advances are more common at the end of subjective night (from CT 20 to CT 24) and in the beginning of subjective day (from CT 0 to CT 6), while phase-delays are restricted to early and middle subjective night (from CT 12 to CT 20).

Statistical analysis confirms that phase-shift responses are significantly different in time, as judged by the data separated in 4 blocks: CT3-CT9, CT9-CT15, CT15-CT21 and CT21-CT3 (Kruskal-Wallis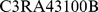,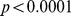). Wilcoxon comparisons of the groups, with the Bonferoni correction (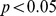), indicates statistical difference between CT3-CT9 and CT9-CT15, CT3-CT9 and CT15-CT21, CT9-CT15 and CT21-CT3, CT15-CT21 and CT21-CT3.

### Computer Simulations

**Figure 4:**
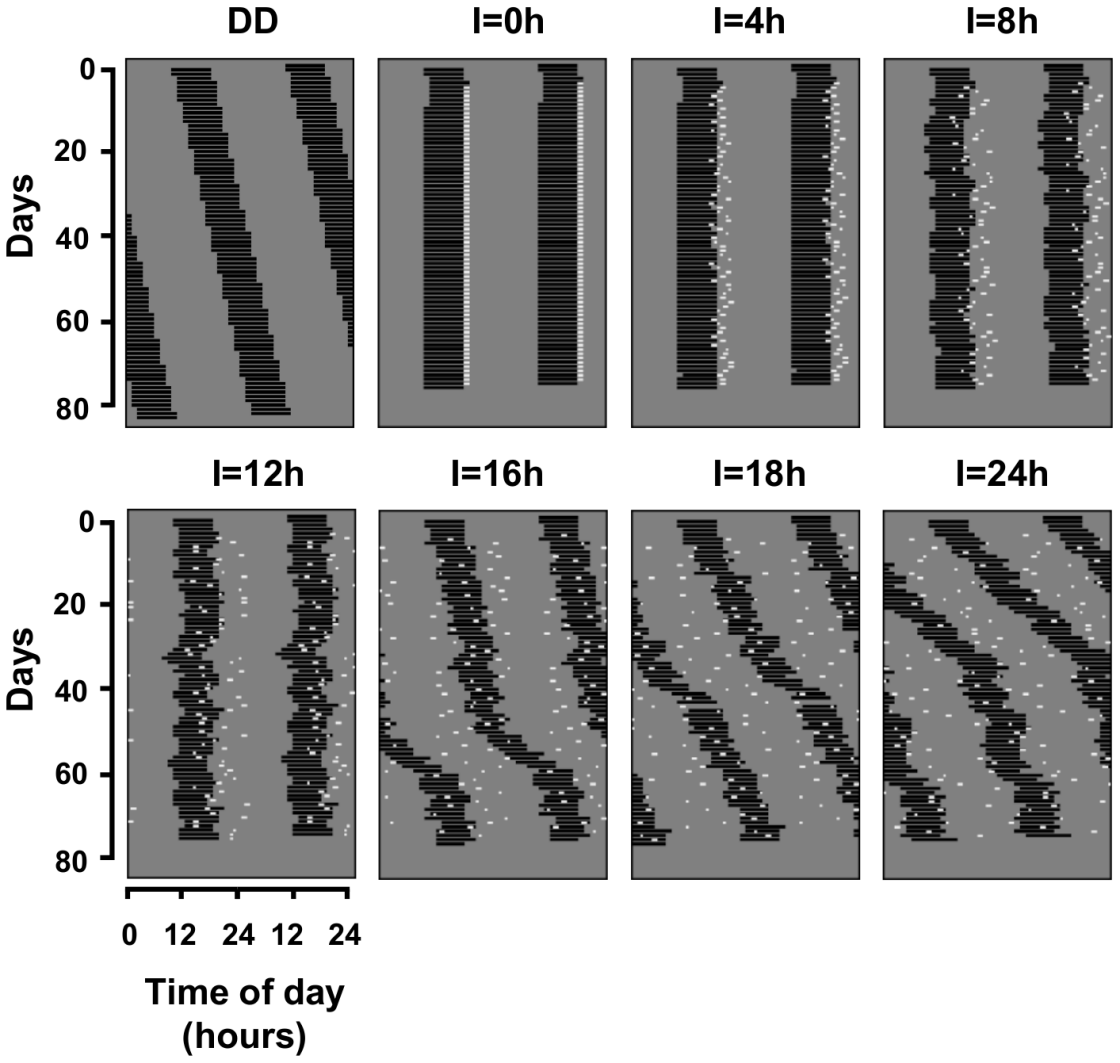
Dynamics of the model oscillator under different simulated light-regimens. The upper-left actogram presents the oscillator in a free-running condition, under a simulated constant darkness (DD). The following graphs show the oscillator under simulated pulse regimens. Except for the DD condition, values over the actograms indicate the duration, in hours, of the time-interval I when the pulses occur. Pulse-times are represented by white dots over the gray background representing a simulated dim background illumination. A consistent 24 h-period is only visualized up to the time-window duration of 12 h. The abscissas for all actograms are the same as indicated under the “I_ = _12 h” graph.

A summary of the results from our computer simulations is presented in the actograms of Fig. 4. Under a simulated DD condition there is no synchronizing cue and the model oscillator free-runs with an intrinsic period greater than 24 hours.

The other actograms show the dynamics of the oscillator under the simulated light-pulse regimens. The **I**_ = _0 h regimen (LD 1∶23) effectively entrains the oscillator, as expected. This mirrors the results obtained experimentally with tuco-tucos (Fig. 2A).

As the duration of the time-interval **I** is progressively increased, pulses become more scattered in time. Due to the extreme simplifications of this light regimen, loss of entrainment was expected to occur as soon as **I** were minimally increased. However, a visual analysis indicates that the pulses sustain entrainment of the model oscillator to a wider extent than expected. For **I**_ = _0, 4, 8 and 12 h, a 24 h-period is visualized (Fig. 4). **I**_ = _16, 18 and 24 h (which represents an unreal 24 h duration light phase) regimens do not sustain entrainment of the model oscillator to a 24 h-period. Periodogram analysis confirmed the visualized tendency (Fig. S3). Nevertheless, this simplified model does not ensure a fixed phase of entrainment between the oscillator and the photophase along increasing **I** regimens. This is indicated by the increasing overlap between the end of activity and the light pulses (Fig. 4, I = 4;8;12). Furthermore, on a day-to-day scale, activity onset phases become progressively more variable in regimens with greater dispersal of the pulses, until the phase-variability reaches a plateau (Fig. 4 and S4).

## Discussion

The present study shows that even the simplest model of the daily light exposure regimen is sufficient to entrain a limit-cycle oscillator that displays a typical circadian PRC. This conclusion indicates that the field entrainment that was indirectly confirmed in our previous studies [3] could be upheld by the peculiar light exposure regimen of this subterranean rodent.

The PRC of the tuco-tucos has essentially the same general features known for epigeous species, with phase-advance regions at the end of subjective night and beginning of subjective day, and phase-delay regions restricted to early and mid subjective night (Fig. 3B). To our knowledge, this is the first PRC of a subterranean animal based on circadian phase shifts, distinctly from circadian c-fos expression [9]. Hence, a single light pulse of only one hour per day at the same time should be sufficient to effectively entrain the circadian system of tuco-tucos, as has been already reported in other rodents [4], [5], [19], [20], and particularly in the subterranean blind mole-rat [21]. Because the free-running period of tuco-tucos is on average longer than 24 h, the shape of PRC predicted that entrainment by T24 single pulse cycles would be attained with light stimuli established at activity offsets, where light stimuli promote phase-advances [19], [20] as demonstrated in Fig. 2A.

Although single pulse T-cycles within the lab are far discrepant from the light exposure patterns that most animals face in the field, it was proposed that they could mimic natural entrainment [22], at least in nocturnal animals that presumably expose to light during twilight hours [19], [23]. In fact, several studies under natural or semi-natural conditions [24]–[33] have highlighted that light exposure is actively controlled by organisms and that some species are only exposed to fractions of the natural LD cycles, and particularly to the twilights [34]–[37]. On the other hand, the complexity and perspectives of studies on natural entrainment were broadened with investigations on diurnal ground squirrels carrying light sensors in the field. Spectacularly, these animals are not exposed at all to light during twilights so that their natural entrainment is achieved “without dawn and dusk” [38].

Previous studies have approached natural entrainment under complex light exposure patterns through mathematical modeling and computer simulations. While Wever [39] has modeled the effects of twilights on entrainment, Beersma *et al* [40], [41] have approached synchronization of diurnal animals and humans [42] to continuous and fluctuating light intensities along the day. Furthermore, Friesen *et al* [34] have modeled the effects of feed-back modulations of varying eye sensitivity on the shape of the PRC.

Our computer simulations intended to verify how random the sporadic light exposure events could be and still be effective for photic entrainment. When modeling a complex phenomenon, it is a common path to start with a minimum model and then add more elements in a systematic way, so that the key factor leading to the investigated mechanism is more easily identified. If the simplest light regimen had failed to entrain, then more realistic features would have been added to the light exposure model, namely more pulses per day, varying light intensity along the day (based on the daily profile shown in Fig. S5), non-uniform randomness, varying pulse durations and varying light frequency spectrum. To our surprise, the simplest random light regimens described in the present paper are already sufficient to entrain a limit-cycle circadian oscillator, at least by ensuring the 24 h periodicity, even when the interval **I** achieves large values, consistent with the range of natural photophases. This is indeed surprising if we zoom the phenomenon to a daily scale (Fig.1B left), where light pulses are expected each day to instantaneously phase shift the limit-cycle oscillator. For randomly scattered pulses this would lead to very erratic activity patterns. However, if we look at the light regimens over several days (Fig. 1B right and 4) a pattern of day and night emerges and the cyclic dynamics of entrainment becomes more evident. Accordingly, entrainment is lost when **I** becomes too large and day-night is no longer recognizable. Interestingly, the route to entrainment loss due to **I** manipulation seems to be similar to that found when *zeitgeber* periods are changed, based on spectral analysis [43].

Our simulations showed that the limit-cycle oscillator is entrained quite smoothly rather than by abrupt daily phase shifts (Fig. 4 and S4). This smoothness is expected to increase as the relaxation rate [44] of the limit-cycle oscillator (fastness of amplitude recovery) is decreased. Due to the non-instantaneous recovery of the limit-cycle oscillator amplitude after a light pulse (Fig. S6), the timing information of random light pulses is integrated along the entrainment process and the occurrence range is processed as the photophase of the day. Aftereffects and transients displayed by tuco-tucos in our previous studies [1] [3] reinforce the proposition that their circadian oscillators might have low relaxation rates. A similar mechanism of light-time integration was seen when modeling chronic jet-lag regimens, where periodic phase shifts of the LD24 cycle were shown to be processed by the circadian oscillator as a new effective *zeitgeber* with a period different from 24 h [45]. A simplified model of the light regimen experienced by tuco-tucos was used in this work to evaluate entrainment effectiveness. Adjustments to this light regimen model are required to fine-tune the phase of entrained oscillator with respect to the daytime. Notwithstanding, our simulations have shown that the randomly scattered daily light pulses restricted to daytime hours are able to ensure the 24 h periodicity in an otherwise free-running circadian oscillator. Field entrainment of tuco-tucos can thus be upheld by photic entrainment, even through the irregular light exposure that they face on the daily basis [46] [47]. A striking and unexpected prediction of our model is that entrainment may be achieved even if this random light regimen is composed by one single light pulse per day, scattered in daytime hours. This prediction is now being experimentally tested in tuco-tucos in laboratory settings by our group.

## Supporting Information

Figure S1Phase Response Curve of the model Pittendrigh-Pavlidis limit-cycle oscillator for parameters (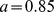, 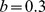, 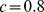, 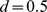). The magnitude and direction of the phase-shift response depend on the relative time (circadian time) of the pulse (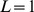).(TIF)

Figure S2 Actograms of the running-wheel rhythms of tuco-tucos used to build the PRC. Numbers over the graphs are lab-identification codes for individual animals. Other specifications as in Fig. 2A.
(TIF)

Figure S3 Period analysis of the rhythms of the model oscillator under the different simulated light-regimens. Black lines represent values of the analysis (Qp) for each period and red lines the significance level (Sokolove and Bushell, 1978) [13]. Under DD the oscillator has a period greater than 24 hours. When subjected to light pulses in regimens from **I**_ = _0 to 14 h, rhythms attain a 24-hour period. For pulses distributed in longer intervals (**I**_ = _16, 18, 24 h) synchronization to the 24 h cycle is lost.
(TIF)

Figure S4 Day-to-day variability of the model oscillator phase under different simulated light regimens. Standard deviation of the oscillator daily activity onsets was used as a measurement of inter-day phase variability. Even though the oscillator sustains a 24 h period in all pulse regimens up to **I**_ = _14 h (Fig. 4 and S2), phase variability increases as simulated light-pulses become more scattered along the day (greater duration **I** of pulse occurrence interval). It is however noticeable that phase variability does not rise in a linear tendency, but rather plateaus, suggesting inertia of the model oscillator phase in spite of great dispersal of pulses along the day. This may reflect the intrinsic characteristic of limit-cycle oscillators of integrating stimuli in the long-term (see discussion).
(TIF)

Figure S5 Illuminance profile of the natural photophase in Anillaco, La Rioja (28° 48′ S; 66° 56′ W; 1350 mts). Each circle is a 3–4 days average of measurements registered during field observations. Measurements were performed with a TM-201 light meter (Tenmars Electronics CO., Taiwan) at the soil level, next to the field enclosure.
(TIF)

Figure S6Motion of a limit-cycle oscillator in response to a brief resetting stimulus. On the simulated day 11, the parameter L (blue line) is increased to 1 and quickly decreased back to the basal level, producing the equivalent to a rapid light stimulus. In response, there is change in the amplitude of the state variable R (red line) of the model oscillator. The variable takes more than one cycle to recover its original amplitude. Parameters values were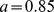, 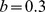, 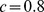, 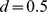. During the stimulus 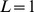.(TIF)
