# Supplementary figures and images for: Modeling Natural Photic Entrainment in a Subterranean Rodent (Ctenomys aff. knighti), the Tuco-Tuco
Source: PLoS One. 2013 Jul 10;8(7):e68243. doi: 10.1371/journal.pone.0068243 (PMC3707898; doi:10.1371/journal.pone.0068243)

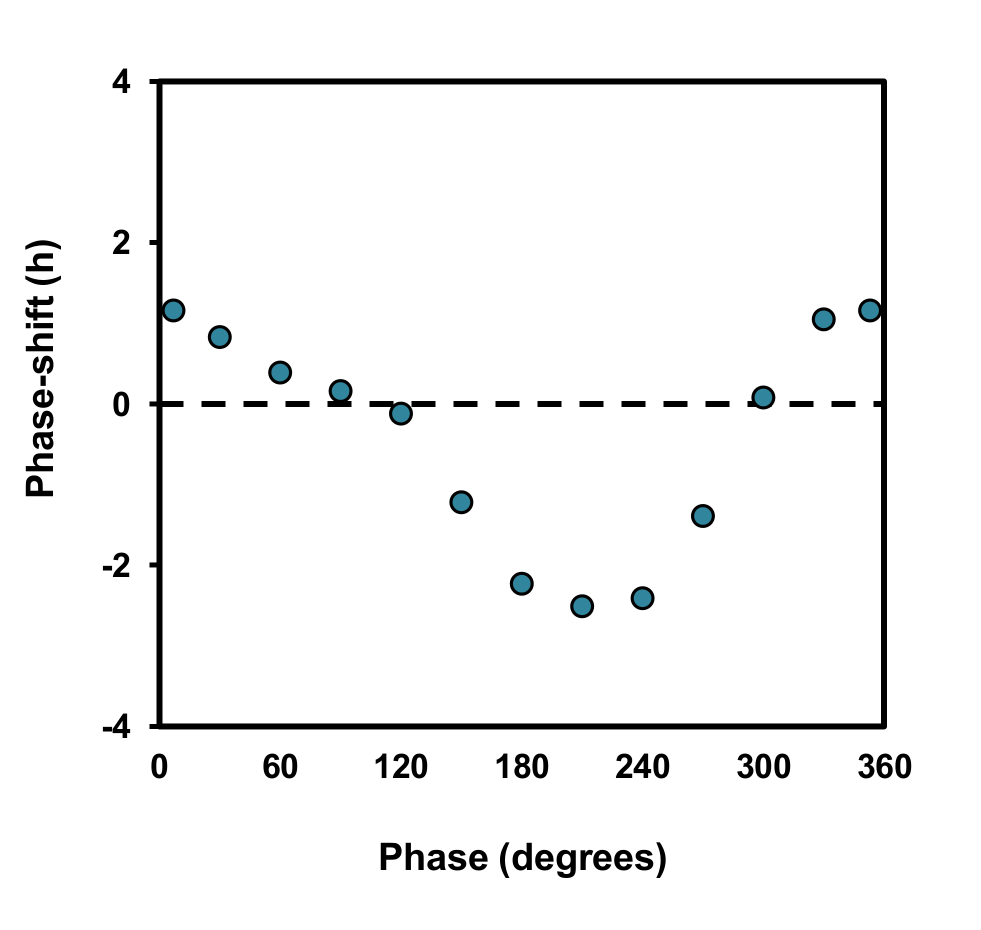

Supplement: Figure S1 — Phase Response Curve of the model Pittendrigh-Pavlidis limit-cycle oscillator for parameters (, , , ). The magnitude and direction of the phase-shift response depend on the relative time (circadian time) of the pulse (). (TIF) [file pone.0068243.s001.tif]

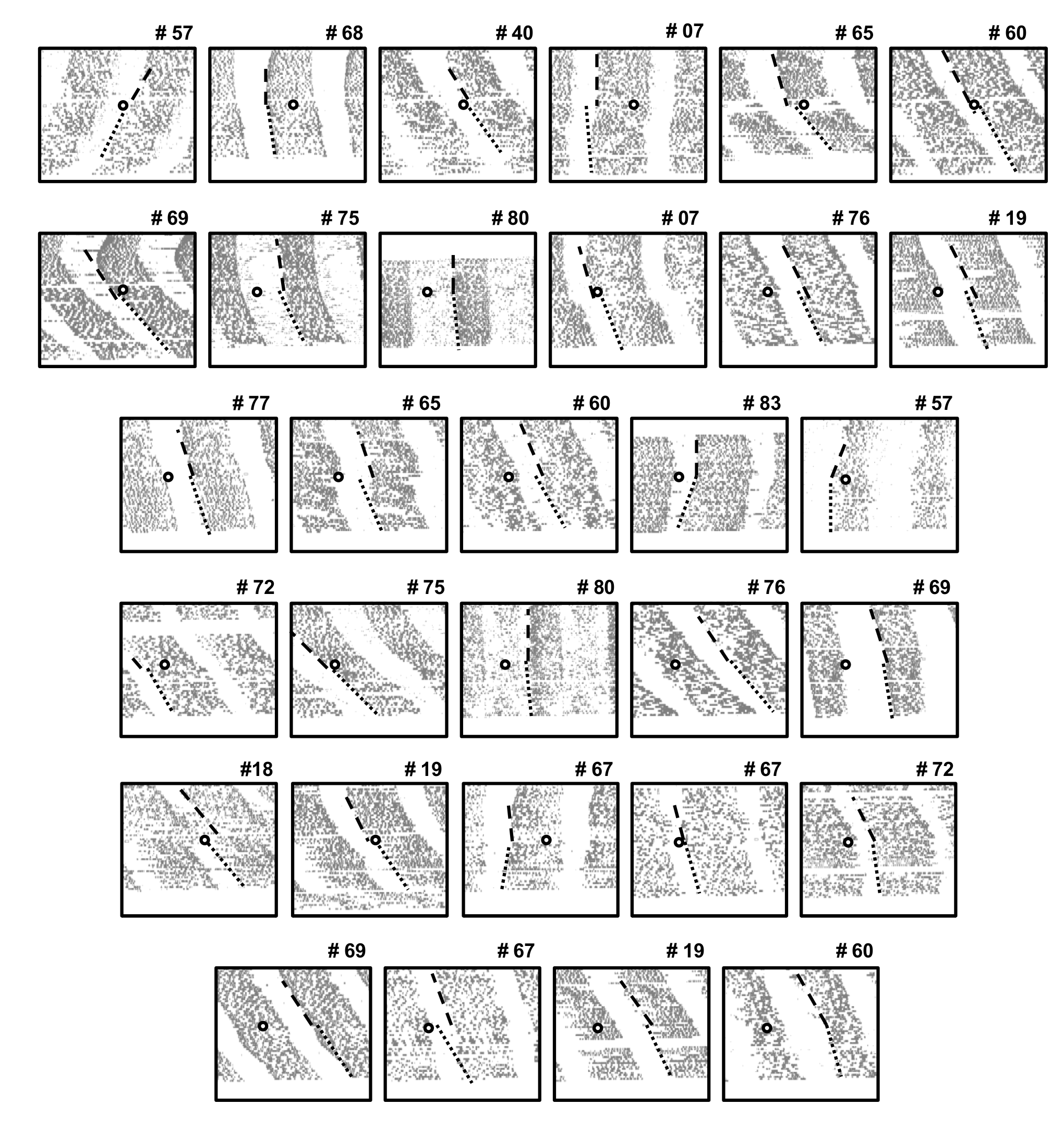

Supplement: Figure S2 — Actograms of the running-wheel rhythms of tuco-tucos used to build the PRC. Numbers over the graphs are lab-identification codes for individual animals. Other specifications as in Fig. 2A. (TIF) [file pone.0068243.s002.tif]

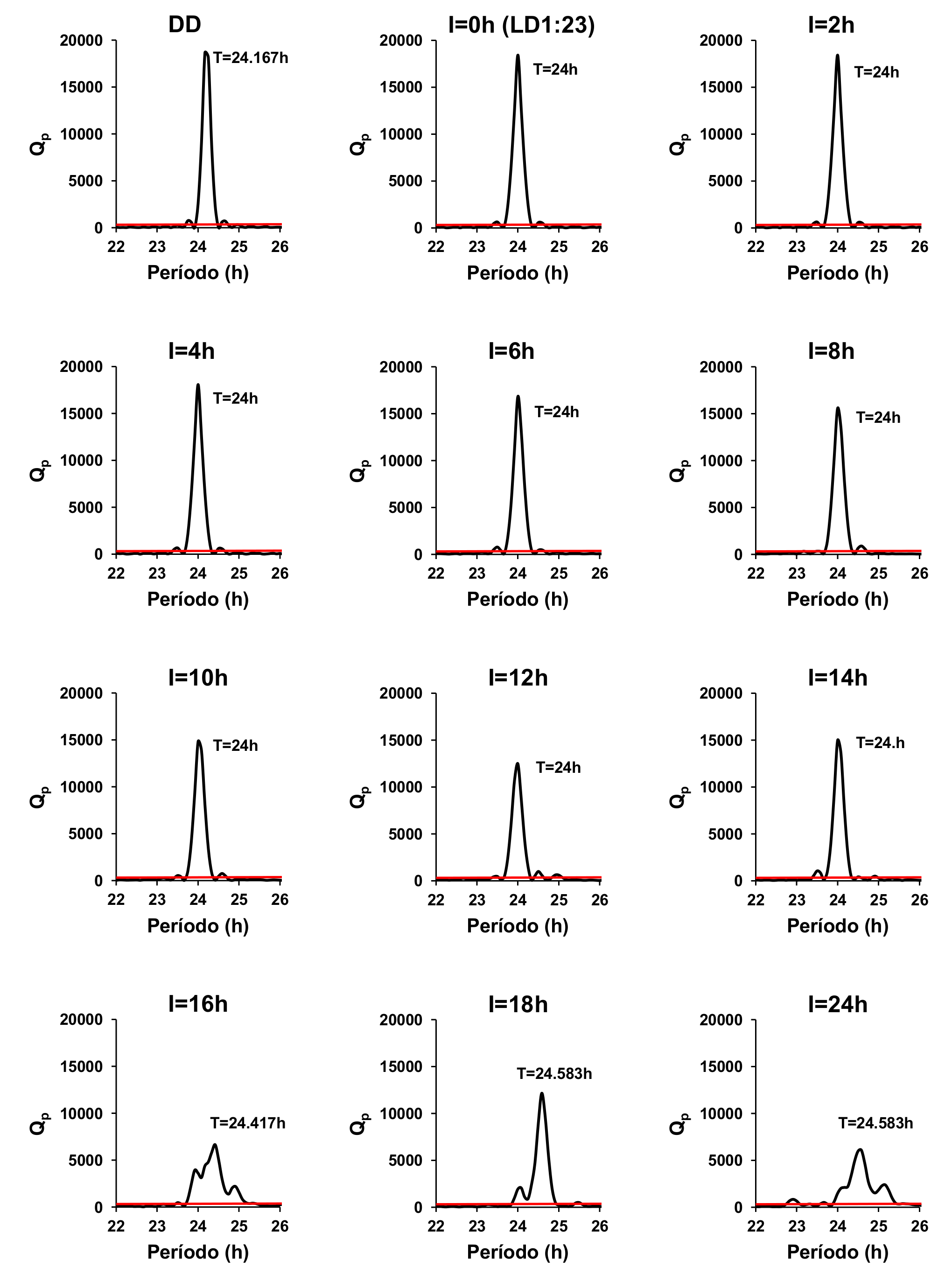

Supplement: Figure S3 — Period analysis of the rhythms of the model oscillator under the different simulated light-regimens. Black lines represent values of the analysis (Qp) for each period and red lines the significance level (Sokolove and Bushell, 1978) [13]. Under DD the oscillator has a period greater than 24 hours. When subjected to light pulses in regimens from I = 0 to 14 h, rhythms attain a 24-hour period. For pulses distributed in longer intervals (I = 16, 18, 24 h) synchronization to the 24 h cycle is lost. (TIF) [file pone.0068243.s003.tif]

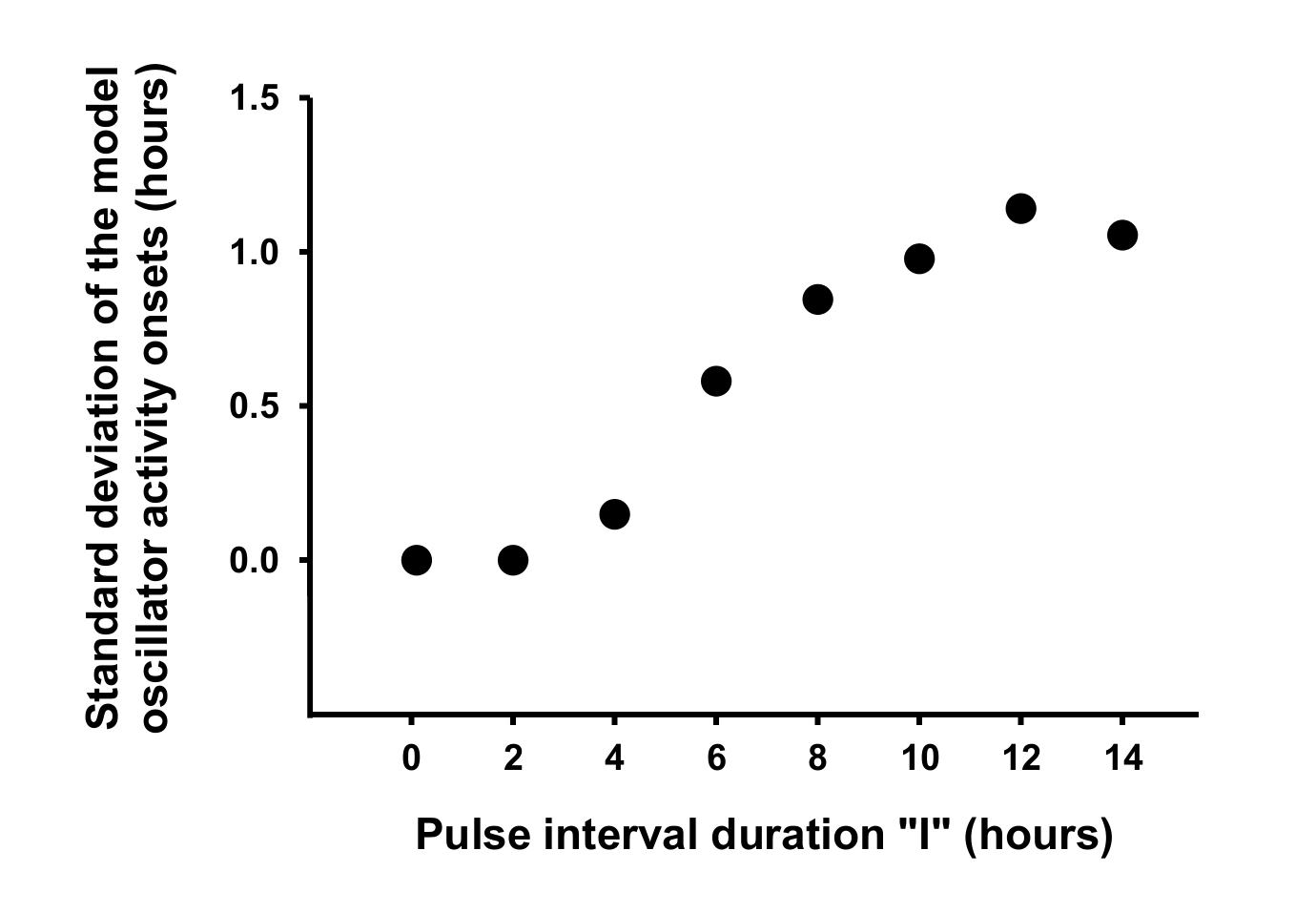

Supplement: Figure S4 — Day-to-day variability of the model oscillator phase under different simulated light regimens. Standard deviation of the oscillator daily activity onsets was used as a measurement of inter-day phase variability. Even though the oscillator sustains a 24 h period in all pulse regimens up to I = 14 h (Fig. 4 and S2), phase variability increases as simulated light-pulses become more scattered along the day (greater duration I of pulse occurrence interval). It is however noticeable that phase variability does not rise in a linear tendency, but rather plateaus, suggesting inertia of the model oscillator phase in spite of great dispersal of pulses along the day. This may reflect the intrinsic characteristic of limit-cycle oscillators of integrating stimuli in the long-term (see discussion). (TIF) [file pone.0068243.s004.tif]

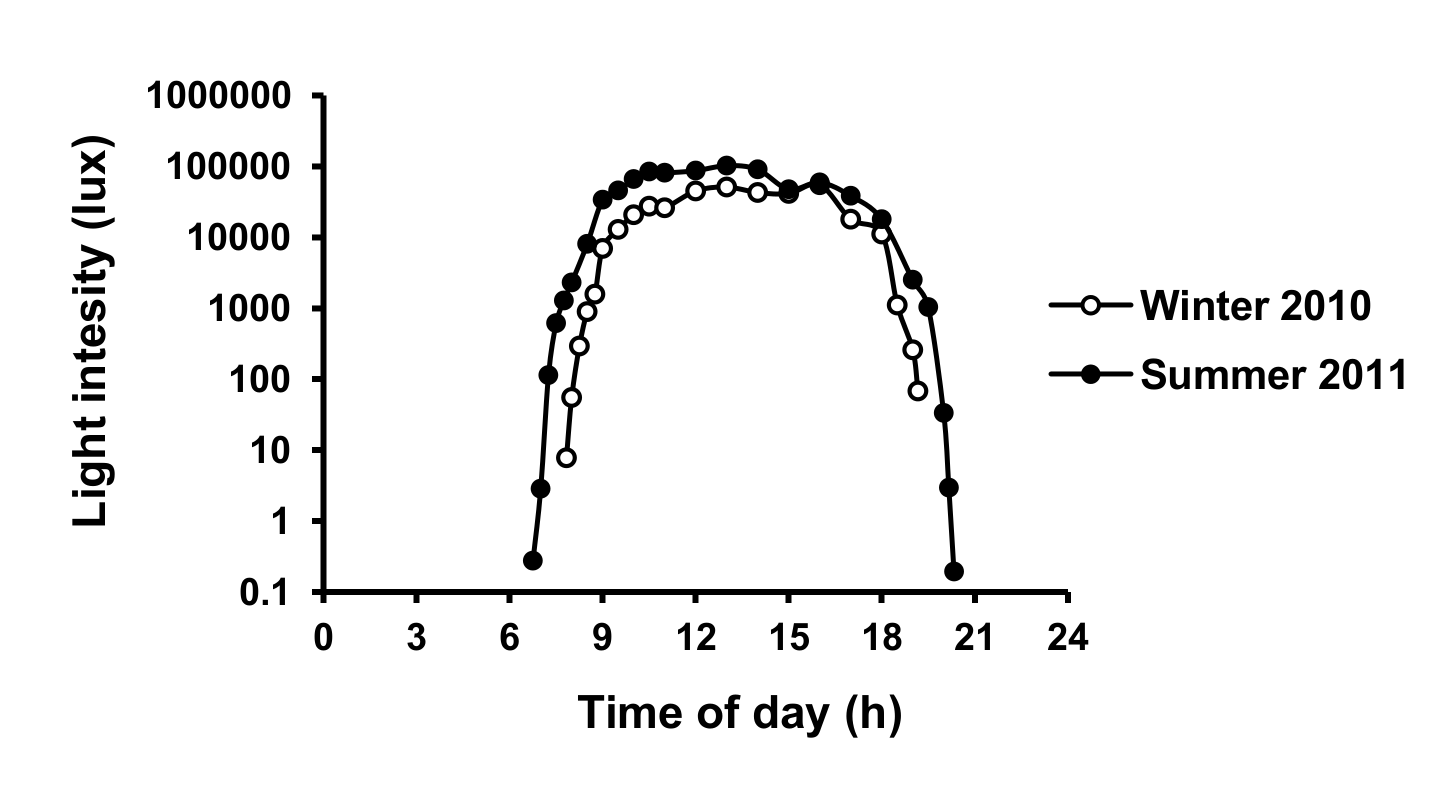

Supplement: Figure S5 — Illuminance profile of the natural photophase in Anillaco, La Rioja (28° 48′ S; 66° 56′ W; 1350 mts). Each circle is a 3–4 days average of measurements registered during field observations. Measurements were performed with a TM-201 light meter (Tenmars Electronics CO., Taiwan) at the soil level, next to the field enclosure. (TIF) [file pone.0068243.s005.tif]

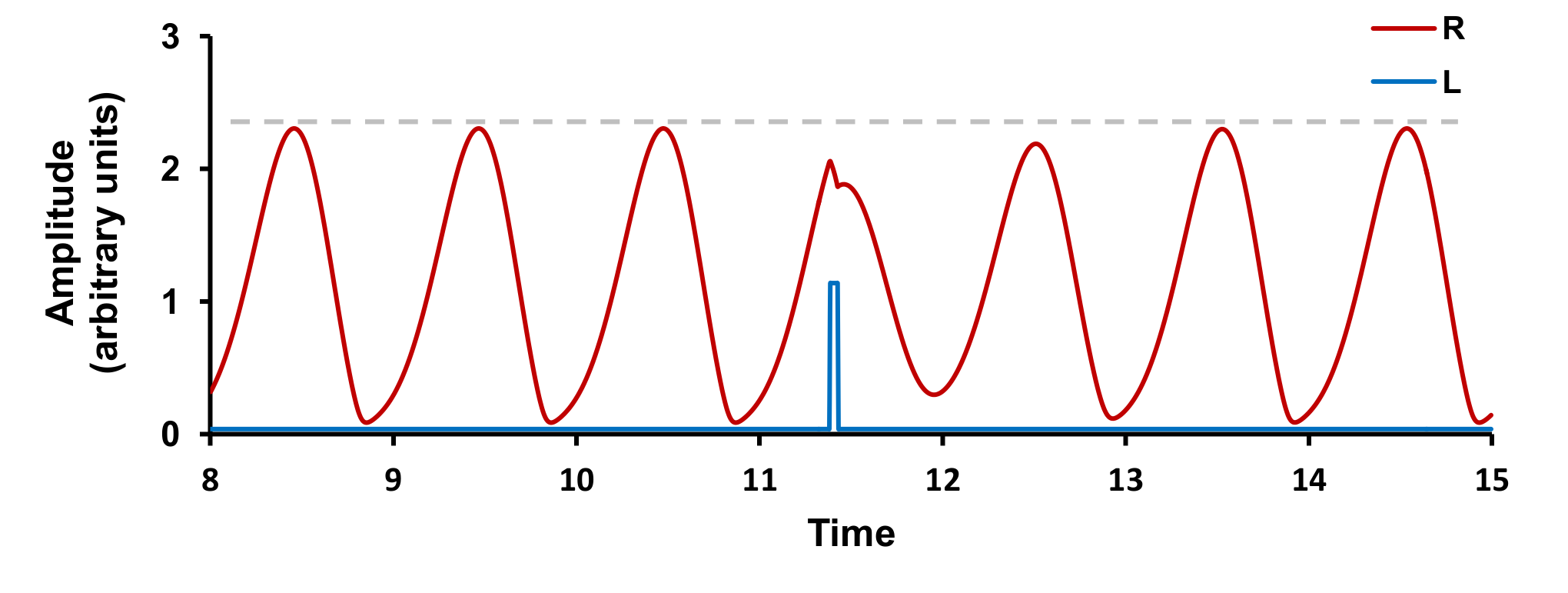

Supplement: Figure S6 — Motion of a limit-cycle oscillator in response to a brief resetting stimulus. On the simulated day 11, the parameter L (blue line) is increased to 1 and quickly decreased back to the basal level, producing the equivalent to a rapid light stimulus. In response, there is change in the amplitude of the state variable R (red line) of the model oscillator. The variable takes more than one cycle to recover its original amplitude. Parameters values were, , , . During the stimulus . (TIF) [file pone.0068243.s006.tif]
